# Risk factors for breast cancer recurrence in postmenopausal women: a bibliometric study

**DOI:** 10.3389/fonc.2025.1522713

**Published:** 2025-04-01

**Authors:** Teodora Hoinoiu, Daniel Piţ, Cristina Oprean, Bogdan Hoinoiu, Andra Diaconescu, Ljubisa Grujic, Magda Mihaela Luca, Daciana Grujic

**Affiliations:** ^1^ Department of Clinical Practical Skills, “Victor Babes” University of Medicine and Pharmacy Timisoara, Timisoara, Romania; ^2^ Center for Advanced Research in Cardiovascular Pathology and Hemostaseology, “Victor Babes” University of Medicine and Pharmacy Timisoara, Timisoara, Romania; ^3^ Doctoral School, “Victor Babes” University of Medicine and Pharmacy Timisoara, Timisoara, Romania; ^4^ Department of Oncology, ONCOHELP Hospital Timisoara, Timisoara, Romania; ^5^ ANAPATMOL Research Center, “Victor Babes” University of Medicine and Pharmacy Timisoara, Timisoara, Romania; ^6^ Department of Oral Rehabilitation and Dental Emergencies, Faculty of Dentistry, “Victor Babes” University of Medicine and Pharmacy Timisoara, Timisoara, Romania; ^7^ Interdisciplinary Research Center for Dental Medical Research, Lasers and Innovative Technologies, "Victor Babes" University of Medicine and Pharmacy, Timisoara, Romania; ^8^ Faculty of Management in Production and Transportation, Management Department, Politehnica University, Timisoara, Romania; ^9^ Dr Grujić Aesthetics, Vršac, Serbia; ^10^ Department of Pediatric Dentistry, Faculty of Dental Medicine, “Victor Babes” University of Medicine and Pharmacy Timisoara, Timisoara, Romania; ^11^ Department of Plastic and Reconstructive Surgery, “Victor Babes” University of Medicine and Pharmacy Timisoara, Timisoara, Romania

**Keywords:** postmenopausal breast cancer, breast cancer risk factors, menopausal women, breast cancer recurrence, bibliometric analysis

## Abstract

Breast cancer is a significant healthcare challenge, and despite advancements in treatment, the risk of recurrence remains a critical concern, particularly for postmenopausal women. Understanding the factors that contribute to this risk is essential for improving monitoring and prevention strategies, ultimately enhancing long-term care and disease management for this patient population. The study analyzes scholarly literature on recurrence patterns in postmenopausal Caucasian women with prior breast cancer, highlighting the potential for innovative insights to reduce breast cancer mortality and improve long-term survival. We used R software and the “R-Bibliometrix” package to analyze postmenopausal breast cancer recurrence. Data was collected from the Web of Science Core Collection database to identify relevant documents and highlight significant collaborative efforts and commonly used terminology. The extensive analysis included 500 articles authored by 3,204 individuals from 195 distinct sources, all published between 2010 and 2024. It specifically focused on assessing the risk of breast cancer recurrence in postmenopausal women. The results underscored several critical factors influencing the risk of recurrence, encompassing hormonal factors, lifestyle influences, the effectiveness of various types of adjuvant therapy, and the role of genetic factors. In conclusion, the research highlights the multifaceted nature of factors contributing to breast cancer recurrence in postmenopausal women. We believe that this study not only enhances the current understanding of the risk of breast cancer recurrence in postmenopausal women but also provides clear directions for future research and improvements in clinical practice and health policy.

## 1 Introduction

Breast cancer remains a major global health concern, ranking among the leading causes of cancer-related morbidity and mortality. Its high incidence and significant risk of recurrence make it a particularly pressing issue in oncology (1–3). Among postmenopausal women, breast cancer presents unique challenges due to hormonal fluctuations that influence disease progression and treatment outcomes (1–3). Addressing this public health burden necessitates a comprehensive understanding of risk factors, clinical challenges, and research gaps to enhance patient care and improve survival rates (4, 5).

Breast cancer is one of the most prevalent malignancies worldwide, with incidence rates rising sharply with age (6–8). Breast cancer has become the most diagnosed cancer globally, surpassing lung cancer, with an estimated 2.3 million new cases. It is the fifth leading cause of cancer-related mortality worldwide. Studies indicate that breast cancer recurrence rates range from 11% to 30% within 5 years and from 20% to 36.8% within 10 years after treatment. Studies indicate that older women face an increased likelihood of aggressive disease phenotypes, with obesity further compounding the risk due to its association with higher estrogen levels (9, 10). Postmenopausal women constitute a particularly vulnerable group, as aging and hormonal changes exacerbate the risk of tumor development and recurrence (11–14). Given these factors, targeted research and personalized treatment strategies are crucial to addressing the unique needs of menopausal patients (12–14).

Multiple risk factors contribute to breast cancer recurrence, including tumor biology, hormone receptor status, lymph node involvement, and molecular subtypes (4, 5). Postmenopausal women face an elevated risk due to a combination of intrinsic (genetic predisposition, aging) and extrinsic (lifestyle, obesity, previous treatment modalities) factors (13, 14). Disease recurrence, whether local or distant, poses a significant challenge to long-term survival, necessitating a deeper exploration of its predictors and management strategies (3, 5, 13).

Despite advances in breast cancer treatment, several challenges persist. The heterogeneity of the disease complicates prognostic assessments, and gaps remain in understanding how age and menopausal status influence recurrence risk (4, 13). Conflicting evidence exists regarding the impact of age on prognosis, with some studies suggesting poorer outcomes in younger women due to aggressive disease subtypes, while others argue that disease characteristics and treatment responses are the primary determinants of survival (3, 13). Additionally, current research often lacks a comprehensive, global perspective on recurrence patterns among postmenopausal patients, highlighting the need for more robust epidemiological and clinical investigations (15–17).

Bibliometric analysis is an emerging methodology for evaluating research trends, scientific impact, and knowledge dissemination in specific medical fields. Bibliometrics is a tool used to investigate the development and structure of knowledge in a specific field of research, and academics have increasingly applied this technique (18, 19). By analyzing scholarly literature on breast cancer recurrence in postmenopausal women, this study aims to uncover influential research patterns, collaboration networks, and citation trends.

This study seeks to address a critical gap in breast cancer research by conducting a bibliometric analysis of recurrence patterns in postmenopausal women. By systematically reviewing existing literature, it aims to provide valuable insights into the evolution of knowledge in this domain, guiding future research efforts and informing clinical decision-making. Given the significant burden of breast cancer among menopausal women, this work has the potential to refine prognostic models, improve treatment personalization, and ultimately enhance patient outcomes.

## 2 Methods

The bibliometric analysis was conducted using the widely recognized R software for its analytical capabilities. To evaluate both the quantity and quality of publications, a detailed analysis was performed through the “R-Bibliometrix” package. This analysis involved exploring various indicators, based on the R Shiny package. The bibliometric data regarding the study of the relationship between postmenopausal breast cancer - and risk factors associated with recurrence were collected from the Web of Science Core Collection database, known for its reliability and consistency. Furthermore, due to its high-quality indexing, the Web of Science is widely considered a precise and in-depth resource for scientific research and evaluation. To identify the most relevant documents regarding the recurrences among postmenopausal women with prior breast cancer, appropriate criteria, search topics, and the most significant keywords were utilized. We compiled relevant articles within our domain using the RStudio software. This examination enabled us to identify commonly used terminology and highlight significant collaborative sorts in particular countries. As the Biblioshiny package only considers articles in English, we retained the terms in this language. The bibliometric data were sourced from the Web of Science database, which was deemed the most relevant and comprehensive for bibliometric analysis focused on recurrences among postmenopausal women with a prior history of breast cancer. Following Lobonţ et al. (2020), Lobonţ et al. (2021), Mustea et al. (2021) and Costea, et al. (2022) were the researchers who employed using similar methodological credentials (20–23). Thus, it was essential to conduct a pertinent analysis to identify and analyses the different perspectives and implications of recurrences among Caucasian postmenopausal women with prior breast cancer. The rigorous review followed specific inclusion and exclusion criteria for potential papers. This led to the selection of relevant articles for the proposed research topic: postmenopausal breast cancer - risk factors associated with recurrence. The data used in this process were obtained from the Web of Science - Core Collection. An essential step in starting this analysis involved the application of filters using keywords: (“postmenopausal breast cancer”*), (“breast cancer risk factors”) and (“breast cancer recurrence”). The initial search yielded 1515 articles related to our research topic. To ensure the material’s relevance and availability, we included only articles published in English. By restricting the time frame to 2010-2024, focusing on the European Union region, and considering only journal articles, we arrived at a final set of 500 articles that were deemed pertinent to our study’s specifications. Therefore, the initial dataset of 1515 articles was narrowed down to 500 through a series of strategic steps to ensure relevance and quality. Filters for specific keywords, English language, and a publication time frame, were applied to focus on studies directly related to postmenopausal breast cancer recurrence. Additionally, restricting the dataset to peer-reviewed journal articles and ensuring a focused citation topic further ensured academic rigor and relevance. These steps culminated in a robust dataset, well-suited for bibliometric analysis on the topic.

**Figure 1:**
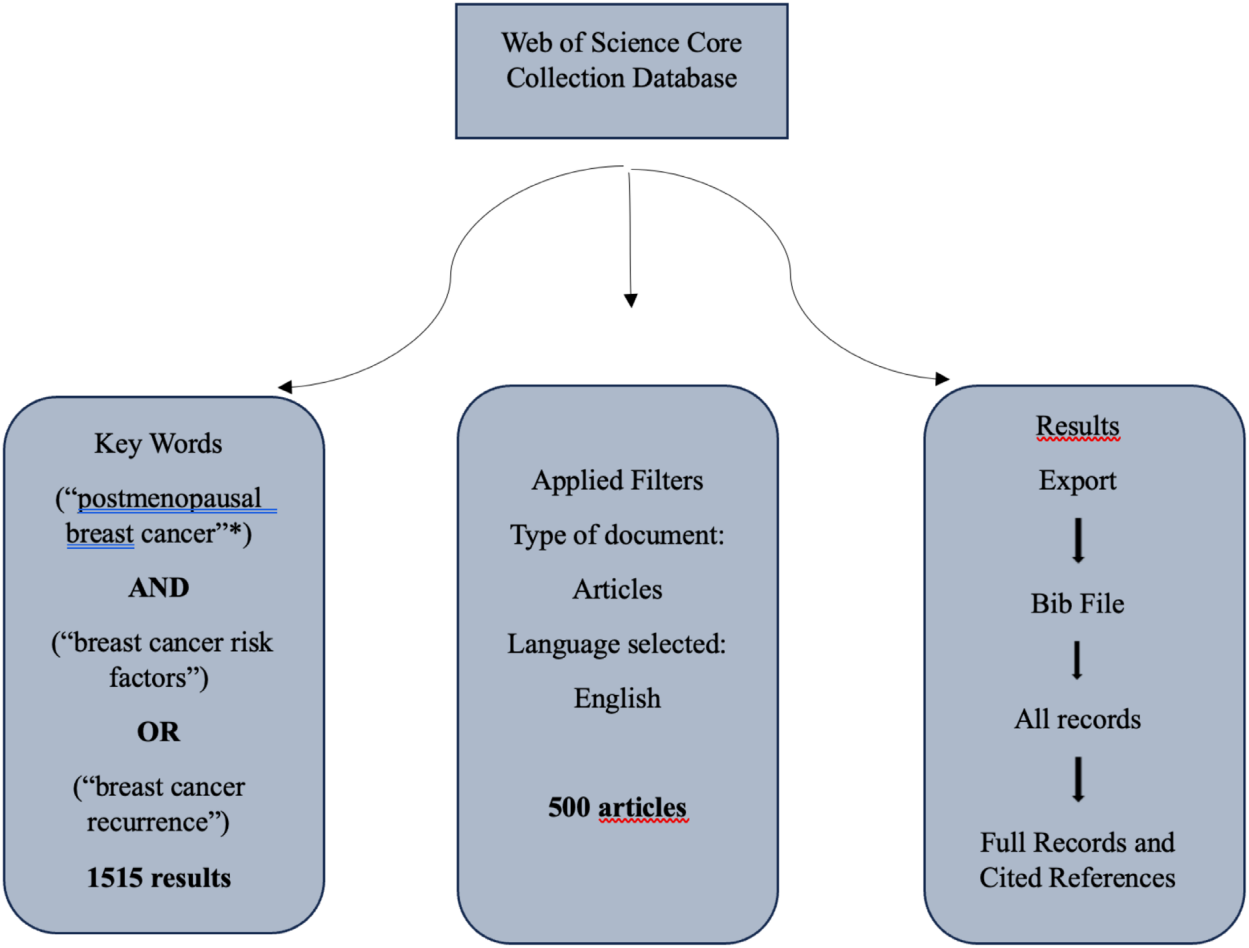
Data extraction from the WoS core collection database. Source: data processed by the author.

To thoroughly explore the key concepts, we meticulously gathered a range of documents, specifically “articles,” spanning from 2010 to 2024, utilizing the Web of Science database. This comprehensive search within the Web of Science Core Collection yielded a total of 500 documents. Following this data collection phase, we proceeded to save and post-process these documents using RStudio software to facilitate a detailed analysis. The methodology for extracting these articles to develop bibliometric networks is illustrated in **Figure 1**, showcasing the step-by-step process involved. This approach not only allowed for a robust examination of the literature but also enabled the visualization of interconnected research trends and themes over the specified period.

The analysis is based on a total of 500 documents written by 3204 different researchers and distributed in 195 sources during the period 2010-2024. Some of these papers have been cited in the Web of Science database. Furthermore, the academic literature exploring breast cancer risk contains a collection of 926 keywords by multiple authors.

Consequently, the bibliometric indicators indicate an average number of approximately 14821 references. This section provides an overview of the characteristics of the scientific literature related to the investigation of recurrences among women with prior breast cancer, according to **Table 1**.

**Table 1:** General data set. Source: own processing.

Selection of relevant data for research	Info
Sources from academic journals, books, and others	195
Documents selected	500
Annual Growth Rate (%)	25.99
Document Average Age	5.62
Average citations per doc	21.79
Total references	14821
Contents of the documents	
Keywords Plus (ID)	1359
Author’s Keywords (DE)	926
Authors distribution	
Authors	3204
Authors of single-authored documents	1
Typology of collaboration between authors	
Documents with a single author	1
Documents with a collective of authors	9.17
Documents with collectives of international authors (%)	32.2
Type of selected documents	
Article	495
Data paper	2
Early access articles	2
Proceedings papers	1

## 3 Results

**Figure 2:**
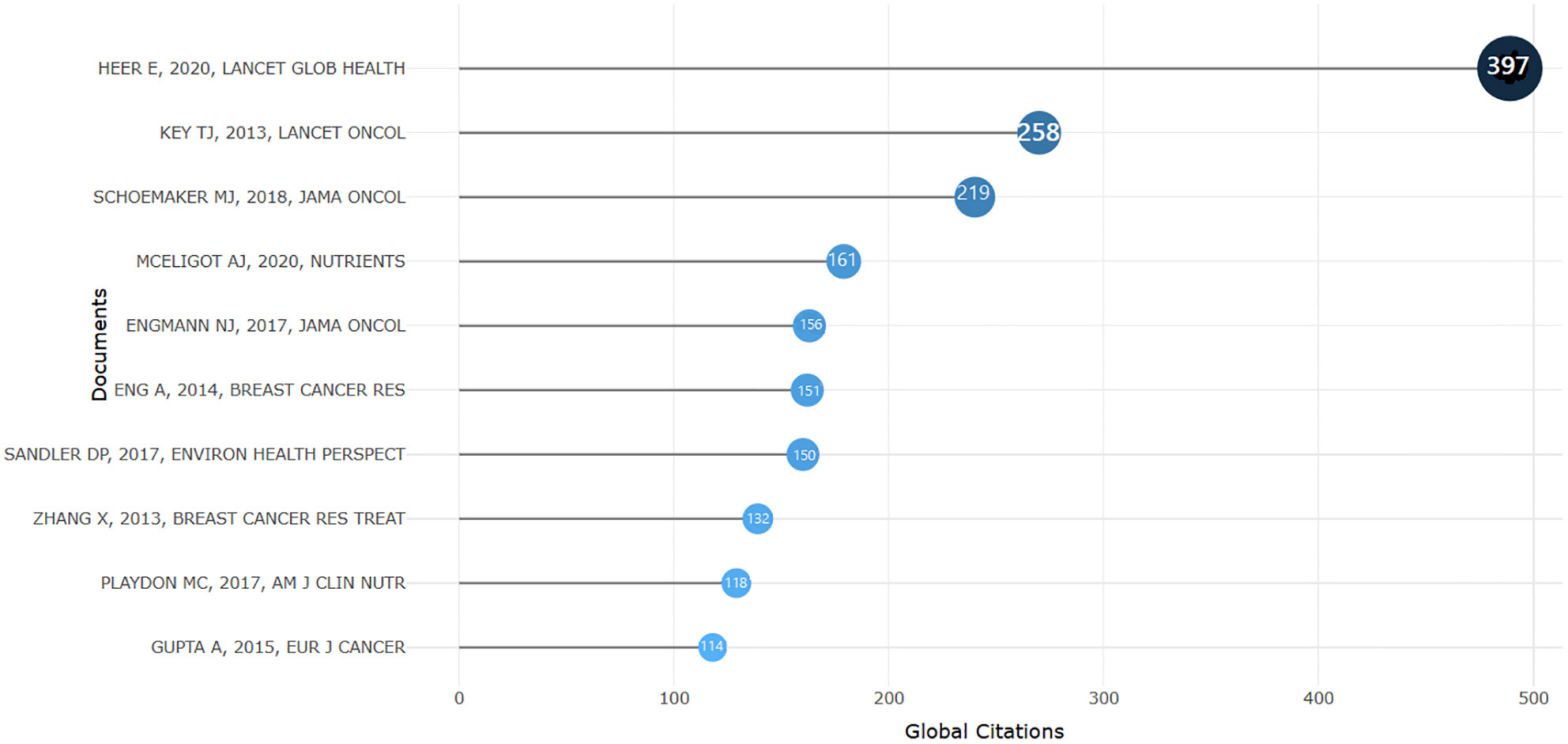
The most cited documents Source: own processing through R program, Biblioshiny application.

The most relevant documents because of the citations observed individually in the period 2010-2024, as follows: the most cited works belong to the author Heer, with a total of 397 citations received, ranking first in the hierarchy, respectively 258 citations for the second document. For the third position, we identified authors such as Schoemaker MJ, who wrote the most cited documents, with a total of 219 received citations. Furthermore, the most relevant document comes from Mceligot AJ, scientific research whose results suggest the relationship between dietary factors and self-reported breast cancer in women aged 50 and older. The analysis identified that high vitamin B12 intake and alcohol use were associated with self-reported breast cancer. The authors suggest that further prospective research is needed to confirm these findings using more recent statistical techniques associated with high expenditures in the healthcare system, with a total of 161 citations received. **Figure 2**, lists the most relevant documents as a result of the citations observed individually in the period 2010-2024.

**Figure 3:**
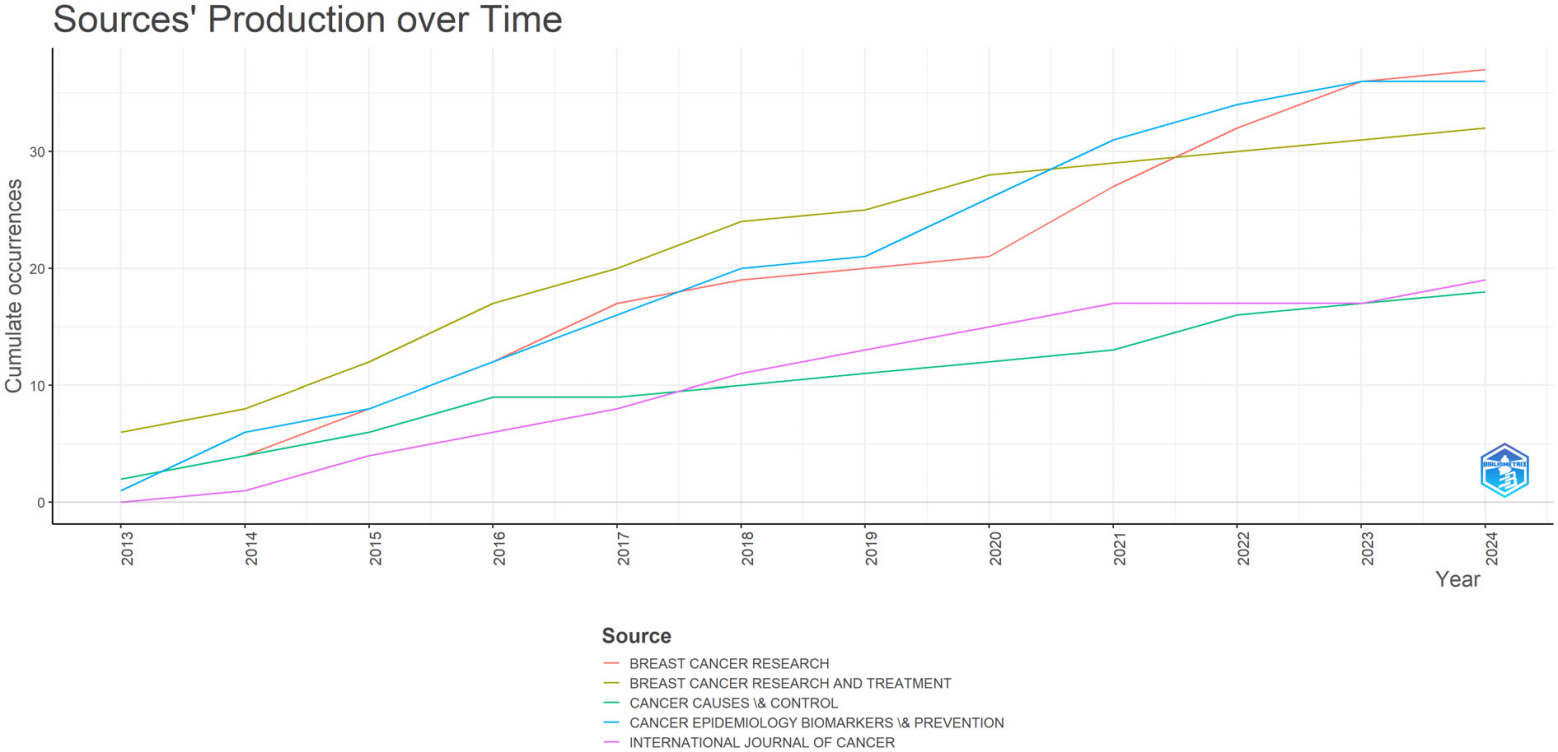
The evolution and dynamics of the sources regarding the number of annual appearances Source: own processing through R program, Biblioshiny application.

Scientific production **Figure 3**, shows the increasing number of annual appearances in the sources, reflecting a growing focus on postmenopausal breast cancer recurrence. This rise is driven by an increased research interest in the topic, enhanced access to research databases and tools, and greater awareness and funding for breast cancer research. The steady growth in publications over time, as visualized through the R program and Biblioshiny application, underscores the expanding attention to this critical area of study. The most relevant sources for the research on the relationship between Locoregional and Distant Recurrences Among Postmenopausal Women with Prior Breast Cancer have been identified based on published documents and citations received over the period from 2012 to 2024.

**Figure 4:**
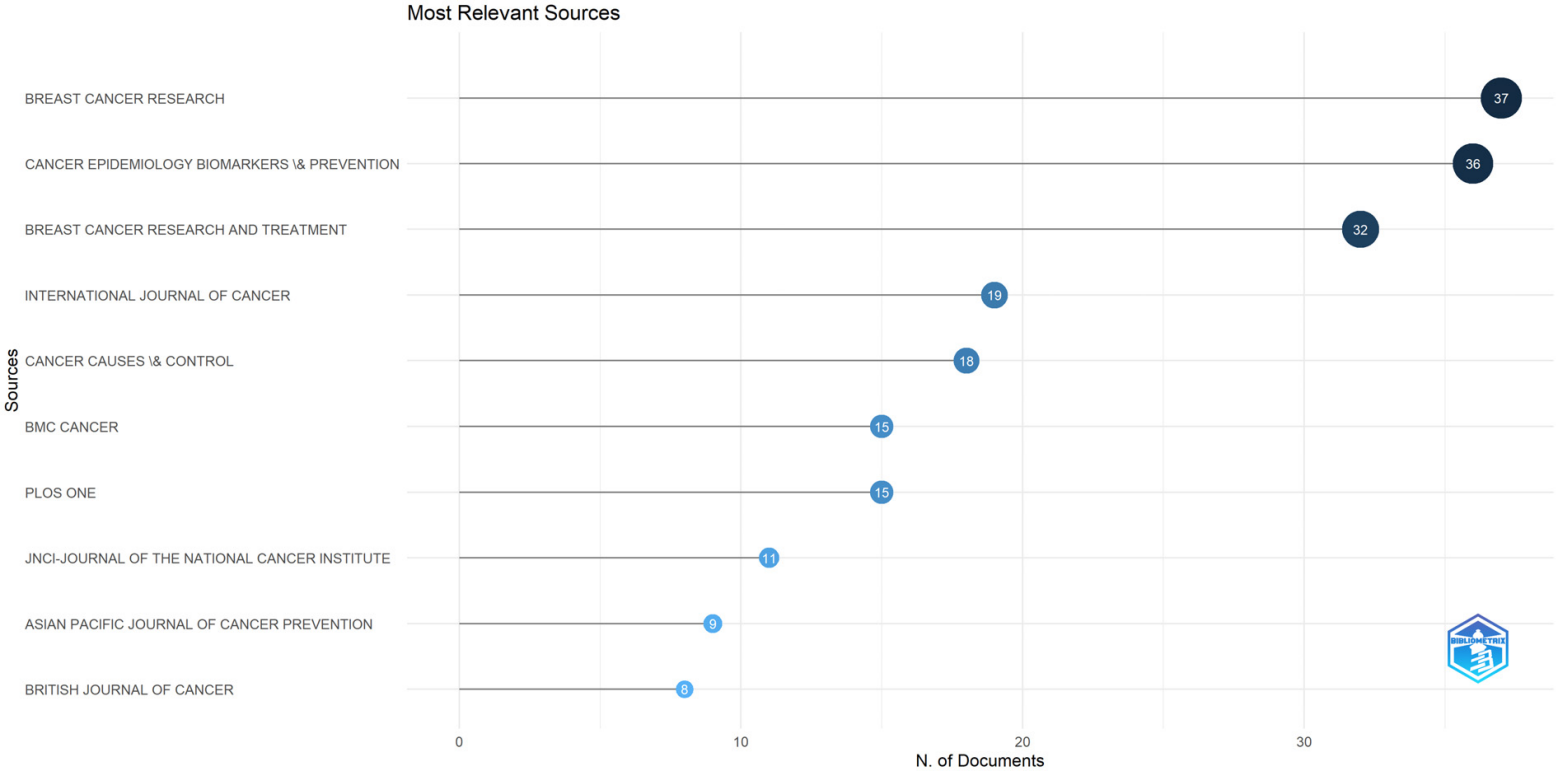
The most relevant sources regarding the number of occurrences. Source: own processing through R program, Biblioshiny application.

Considering the analysis of the level of sources, **Figure 4**, shows us the results of the most productive journals, and a higher level of production of an article in at least one of the years was used to determine the ranking. In the research approach, various sources were analyzed and, in some cases, we noticed that several scientific journals were positioned in the same rank due to the fact that their level of production was the same.

In general, the productivity level of the sources has changed considerably from year to year, in the period 2010-2024, with the number of certain sources increasing significantly in the recently analyzed period compared to the period at the beginning of the analyzed interval.

Regarding the specific scientific sources presented in **Figure 4**, the source “Breast Cancer Research” has the most annual appearances of all publications in the analyzed period (37 annual appearances), followed by “Cancer Epidemiology Biomarkers \& Prevention”, with a total of 37 annual appearances. If each year is considered separately, eight journals had more than nine annual occurrences in the analyzed period, and their annual occurrence accounted for more than half of the annual occurrences in the entire analyzed period. Also, “Breast Cancer Research And Treatment”, “International Journal Of Cancer”, “Cancer Causes \& Control”, and “Bmc Cancer” occupy the main positions in the ranking of annual appearances, all these journals having number more than 15 annual appearances throughout the analyzed time interval. It is noteworthy that the journals “Breast Cancer Research and Treatment”, “International Journal Of Cancer”, and “Cancer Causes \& Control” appeared in the first positions, being the most active and relevant sources in recent years, with increasing annual appearances in the general ranking. Thus, we note that the trend highlights an acceleration of research, but also of significant appearances regarding the incidence of *postmenopausal breast cancer*, with a greater volume of annual appearances in the last years analyzed (2010-2024).

**Figure 5:**
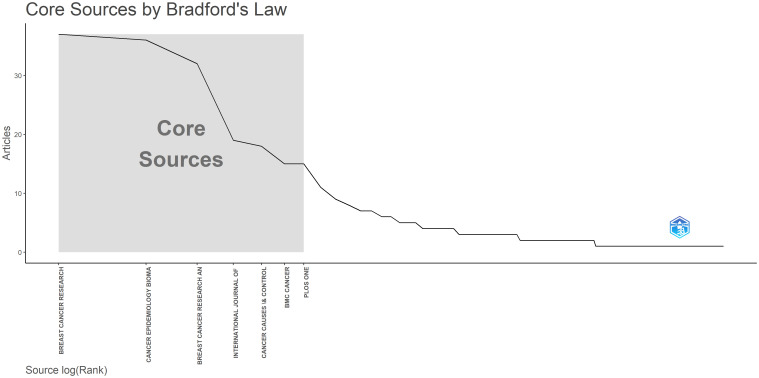
The evolution and dynamics of the sources regarding the number of occurrences Source: own processing through R program, Biblioshiny application.

**Figure 5** illustrates a line graph demonstrating Bradford’s Law. The graph presents the distribution of articles based on the rank of the sources. One of the zones highlights the “Core Sources” - the key sources that publish the most articles, followed by a rapid decline, and then a slower reduction towards the less productive sources. Bradford’s Law is used to identify the central sources in a field and the way in which articles are disseminated across various journals.

Furthermore, this figure, presents the evolution of the sources, and the most significant and prolific can be divided into three types: *(i)* sources that had a low average annual occurrence, but later enjoyed a significant increase and began to have an annual occurrence significant in the last years of the analysis period, with the highest number of occurrences; *(ii)* sources characterized by a notable initial level of annual occurrence, but which significantly reduced their occurrence in the last 15 years analyzed and presented significant intervals of inactivity; *(iii)* sources that started with a relatively low annual occurrence, then showed increasing trends and showed a remarkable and continuous annual occurrence.

**Figure 6:**
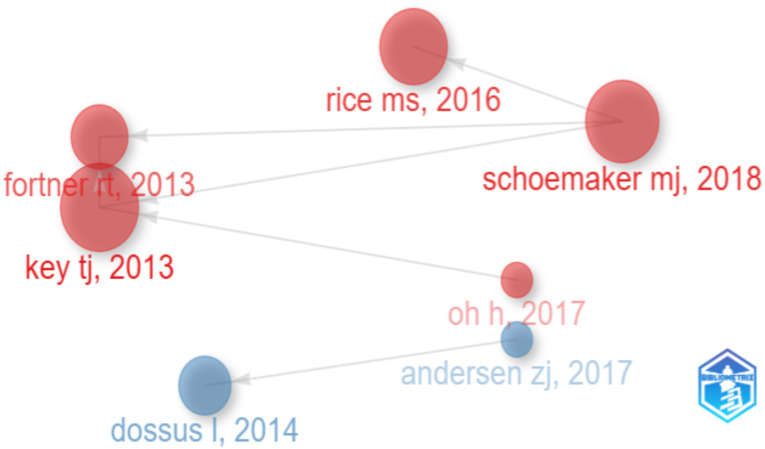
Histograph Source: own processing through R program, Biblioshiny application.

Furthermore, regarding the co-citation network of sources in **Figure 6**, the results highlight the creation of two clusters. In general, the authors located at the extreme boundaries of each cluster indicate a stronger relationship with the other cluster, and the size of the node indicates the level of interaction between the authors, the more visible and larger the node, the more significant the interaction between them. At the same time, it is worth noting that the author “Key TJ” is located in the center of the red cluster, being approximately at a reduced distance from the blue cluster, having strong ties with the other researchers, even with those in the red cluster. Therefore, his position indicates a strong relationship with other authors focused on different scientific approaches and perspectives, but mainly the relationship strengthens most significantly with authors characterized as being part of the red cluster. Author “Andersen Zj” also presents important interactions with the red cluster. Similarly, regarding the red cluster, we identify the researcher “Rice MS” as being located in the center of the red cluster, having the highest connection to journals outside the red cluster, while also showing strong connections to other collaborations within the same cluster.

**Figure 7:**
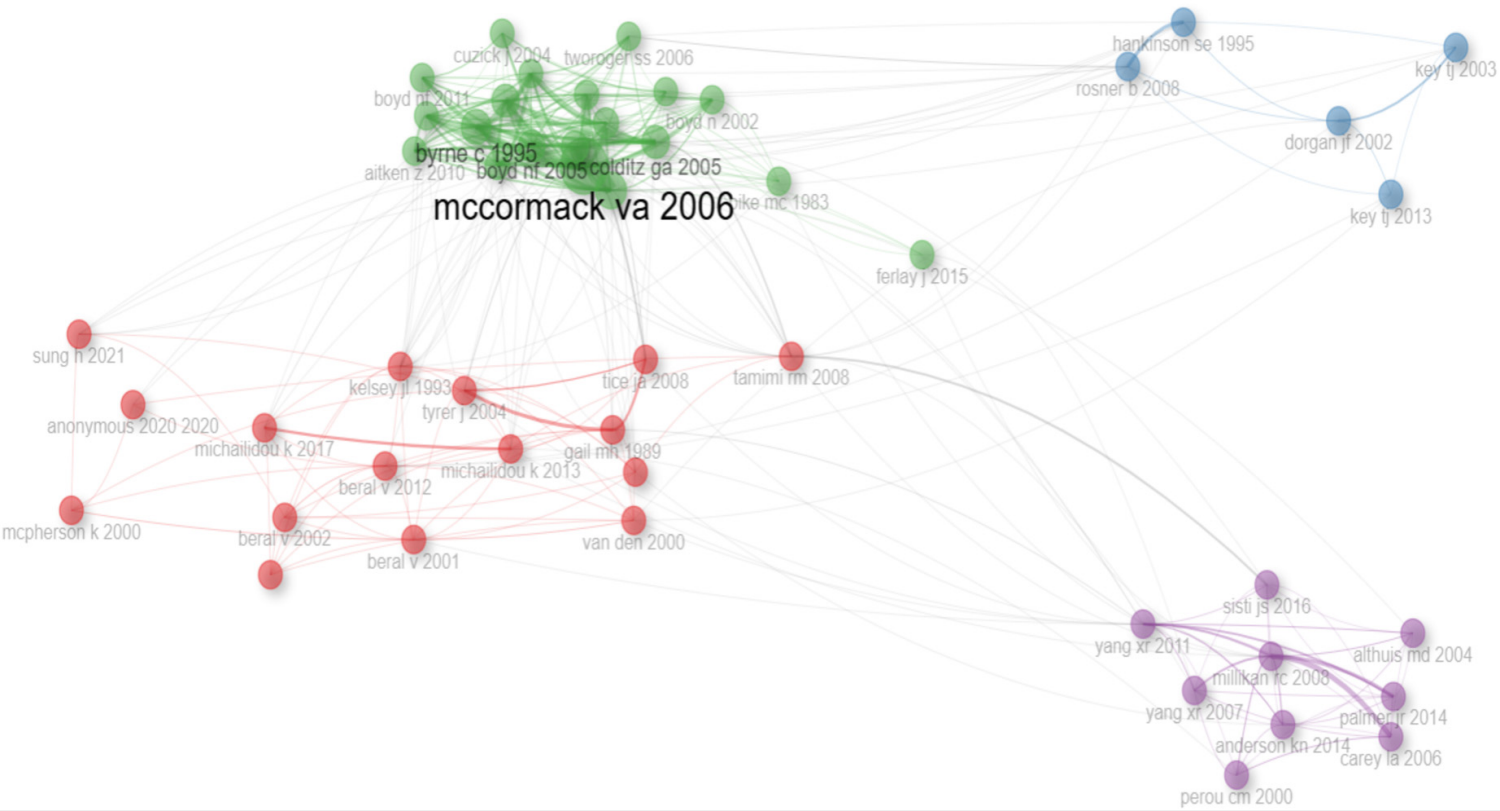
The main collaboration network of authors from the period 2010-2024. Source: own processing through R program, Biblioshiny application.

The author collaboration network depicted in **Figure 7**, reveals four distinct clusters of collaborating authors. Each color in the network represents a group or cluster of authors. These clusters are aligned with the most prolific authors and are numbered in the collaboration network - the red group, the blue group, the green group, and the purple group. The collaboration network showcases the existence of tightly-knit groups that are easily identifiable in the graph, using a threshold of 50 authors. This number of nodes was determined to be the most representative of the expected results of the bibliometric analysis in terms of authors. Employing a larger number of nodes would result in a much greater quantity of individual authors, 70 in this case, rendering the network far more complex and consequently more challenging to comprehend. Regarding the top-ranking authors specifically, they belong to four different numbered groups. The first group is led by the authors “Tyrer J”, “Tamimi RM”, and “Beral V” as the top contributors driving this cluster. Additionally, group 2 is composed of authors such as Dorgan JF, Hankinson SE, Key TJ, and Rosner B as the most connected authors. Finally, authors like Mccormack VA, Boyd NF, Colditz GA, and Pettersson A are identified as part of the purple group.

**Figure 8:**
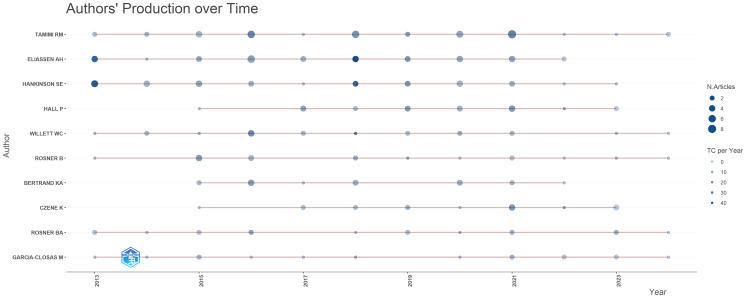
Authors’ production in the period 2010-2024. Source: own processing through R program, Biblioshiny application.

Regarding the analysis of the authors’ production over time, as shown in **Figure 8**, Tamimi RM and Eliassen AH are the authors with the longest track record in Locoregional and Distant Recurrences Among Caucasian Postmenopausal Women with Prior Breast Cancer research. Although their periods of activity were similar, both were at the top of the ranking of the most productive authors. The scientific activity of Burau Viola and Michael Costello is developing in the field that captures Breast Cancer, being the authors with the most outstanding works that have a number of 42, the authors have published numerous studies in this field and are recognized as experts in the analysis of relapses in this category of patients.

**Figure 9:**
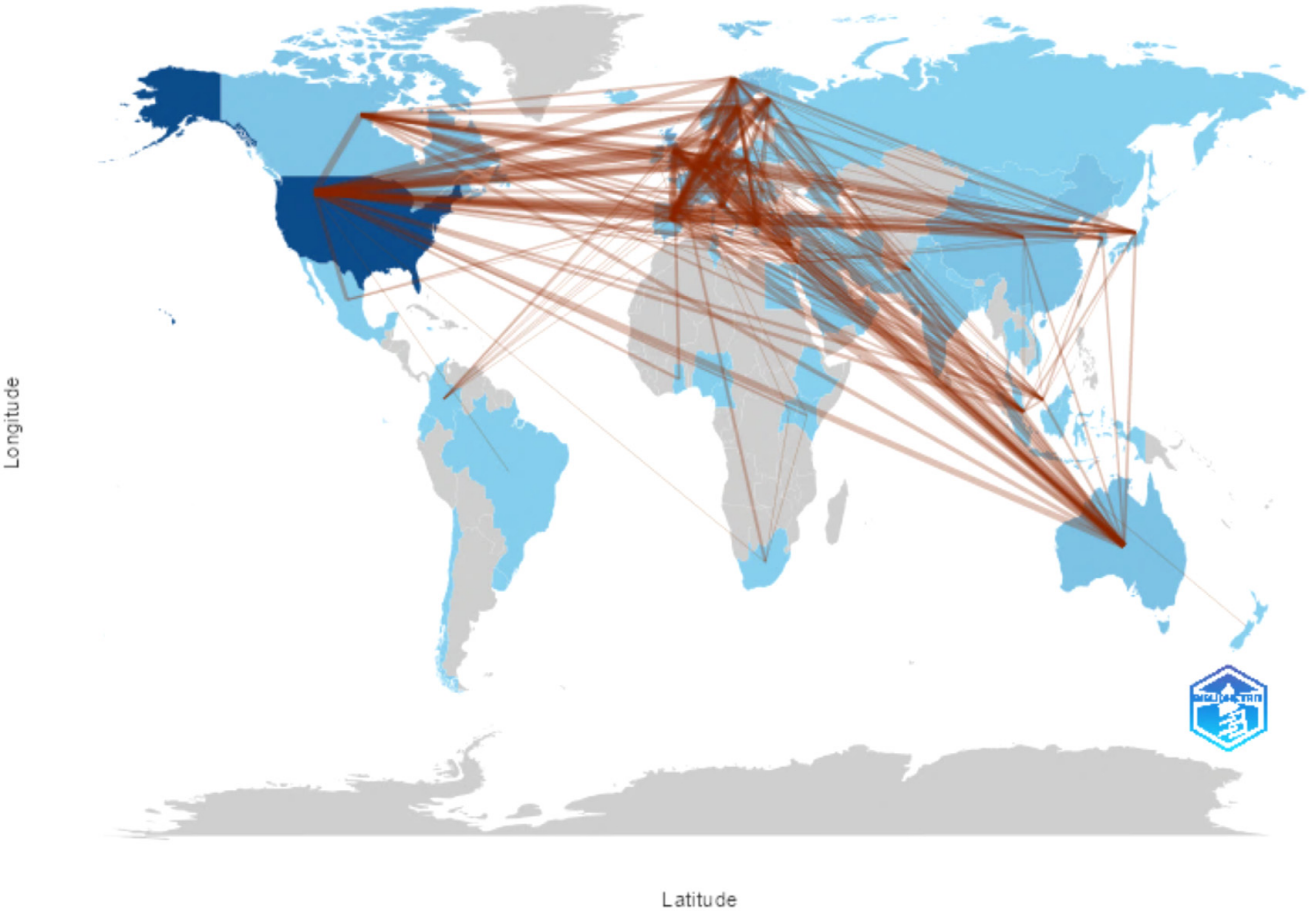
Map of world collaborations on countries prolific in breast cancer research Source: own processing through R program, Biblioshiny application.

The visualization in **Figure 9** demonstrates extensive global collaboration, with a multitude of countries participating in scientific advancement related to postmenopausal breast cancer and risk factors associated with recurrence. The darker hues denote nations with the highest levels of collaborative engagement with others, while the connecting lines signify specific inter-country partnerships. Particularly noteworthy is the significant collaborative linkage between Australia, Canada, the United States, China, South Africa, and the European continent, as well as the high degree of cooperation observed among European Union member states.

**Figure 10:**
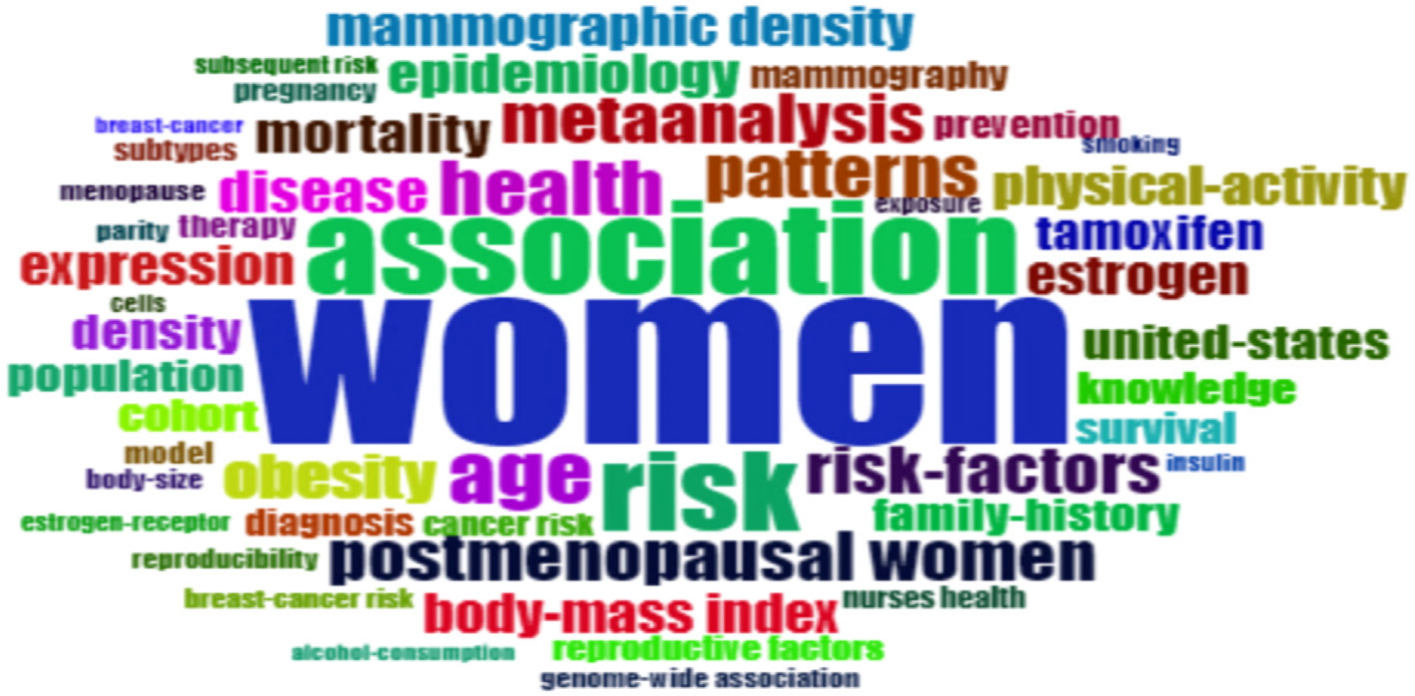
Word Cloud regarding the most important words used in keywords. Source: own processing through the R program, Biblioshiny application.

The terms that appear most frequently in the title of scientific articles are highlighted in **Figure 10**, this presents the unigram word cloud for the entire scientific content sample of papers investigating recurrences among Caucasian postmenopausal women with prior breast cancer. Unigrams consist of a single element in a sequence. The unigrams with the highest visibility are “women” with 120 occurrences, “association” with 64 occurrences, “risk” with 55 occurrences, “age” with 41 occurrences, and “health” with 37 occurrences. Additional unigrams worth noting are “family-history”, “tamoxifen”, “population”, “knowledge”, “mammography”, “prevention”, and “cancer risk”.

**Figure 11:**
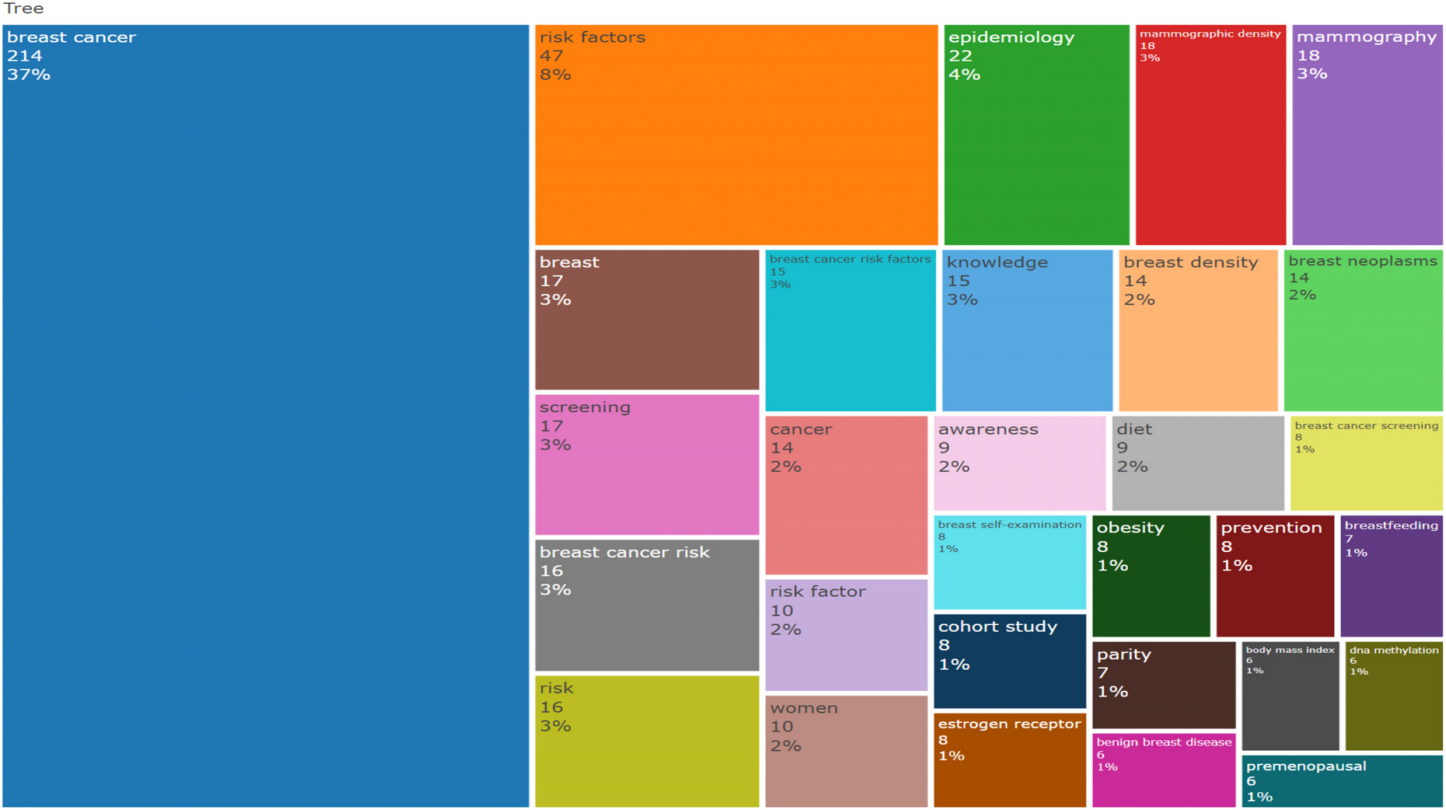
Treemap of the top fifteen words used in terms of frequency. Source: own processing through R program, Biblioshiny application.

**Figure 11**. displays the top 30 most frequent words, revealing the most frequent word combinations, such as “breast cancer” (frequency 37%), “risk factors” (frequency 8%), “epidemiology” (frequency 4%), and with a frequency of 3% we have the words “mammographic density”, “mammography”, “n screening”, “knowledge”, and “risk”.

Themes and thematic areas on the relationship between recurrences among postmenopausal women with prior breast cancer focus on identifying the most relevant themes and research areas.

**Figure 12:**
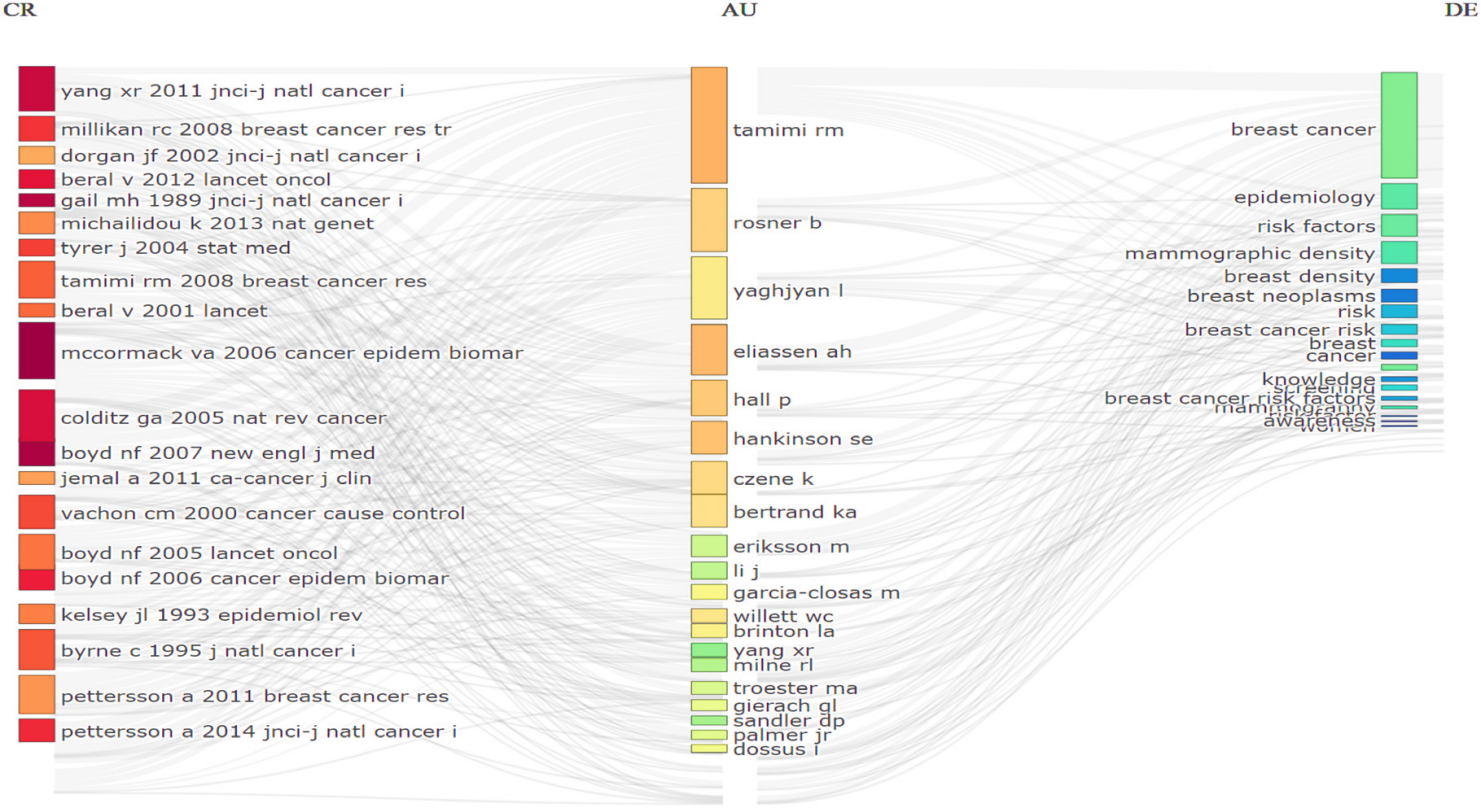
Themes and thematic areas regarding the relationship recurrences among postmenopausal women with prior breast cancer (CR- Cited References, AU- Author and DE-Thematic domain) Source: own processing through R program, Biblioshiny application.

Further, **Figure 12**, presents the diagram of the thematic evolution of the research through two periods previously segmented in 15 years. The thematic evolution provides a global view of changes over time in terms of the specific topics that have been addressed regarding the relationship between postmenopausal breast cancer and risk factors associated with recurrence. Each of the nodes represents a cluster and is labeled by the main authors who address the topic of our theme, as well as by the time intervals. The number of citations of all scientific publications is represented by the size of the corresponding node. Topics in adjacent time slots are connected by rationalizations. The breadth of rationalizations is proportional to the number of citations shared by the connected topics and indicates the relevance between them. The Most Prolific Affiliations Network highlights organizations and institutions with the greatest research impact.

**Figure 13:**
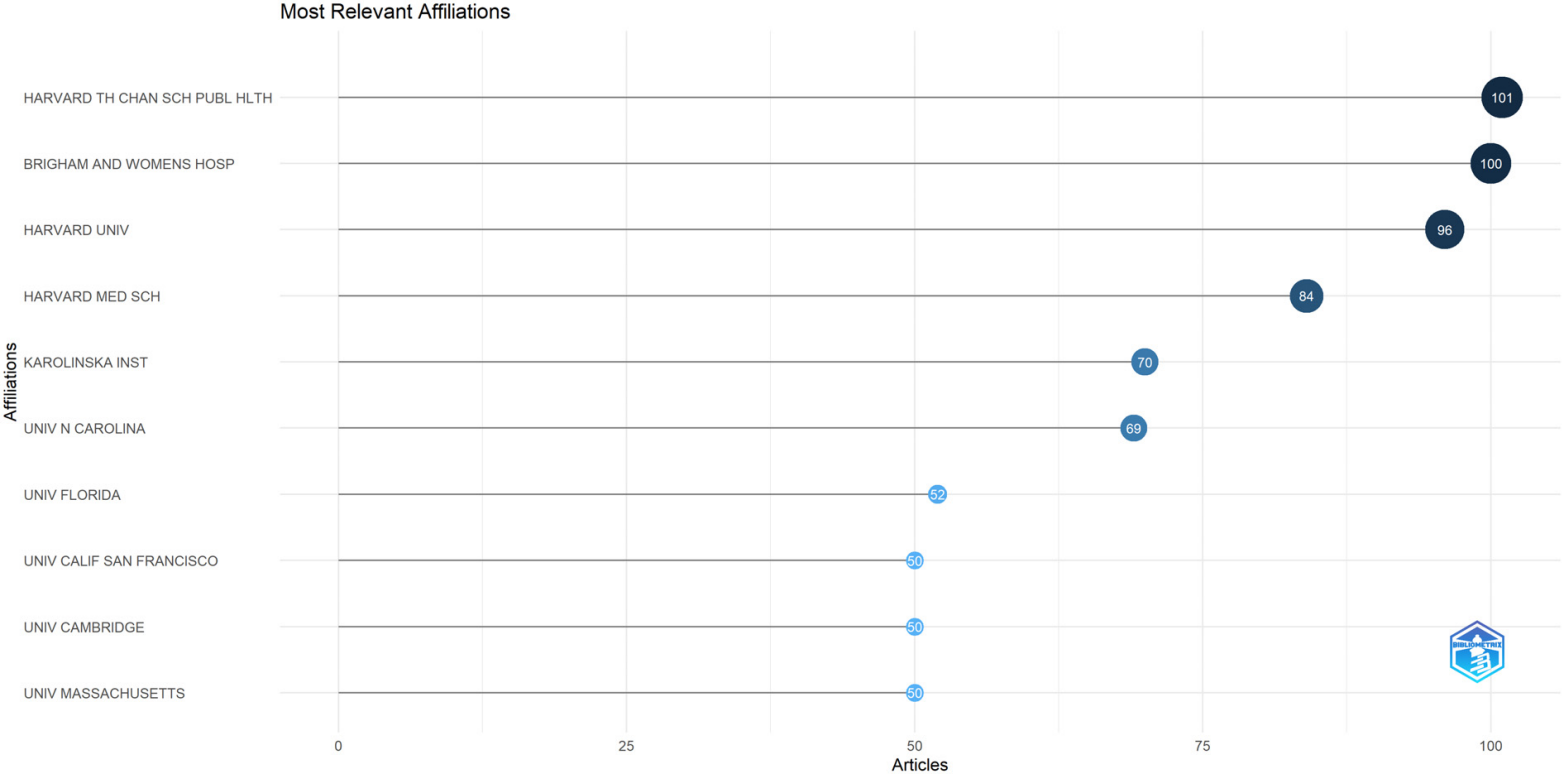
The critical path of the most productive research affiliations, period 2010-2024. Source: own processing through R program, Biblioshiny application.

**Figure 13** showcases the most productive institutional affiliations in the study of the relationship between postmenopausal breast cancer and risk factors associated with recurrence. Notably, Harvard T.H. Chan School of Public Health stands out with 110 relevant publications, followed by Brigham and Women’s Hospital with 100 documents and Karolinska Institute with 70 documents. Furthermore, other prestigious institutions across various countries have also made significant scholarly contributions to this research area, underscoring the global scope and collaborative nature of this subject matter.

## 4 Discussions

Comprehending the risk factors for breast cancer recurrence in postmenopausal women is highly consequential for clinical practice. It is essential for clinicians to be well-informed about these factors in order to provide patients with appropriate counseling and vigilant monitoring, thus reducing the risk of recurrence. Furthermore, encouraging the adoption of a healthy lifestyle and implementing personalized screening programs should be emphasized as crucial components of patient care (11, 24, 25). In managing the risk of breast cancer recurrence among postmenopausal women, education and prevention are pivotal components. It is crucial to empower patients with in-depth knowledge about the diverse risk factors that contribute to recurrence (26). Stressing the significance of regular check-ups, including diagnostic screenings, mammograms, and other preventive measures, is vital for early detection and minimizing the potential for recurrence (27–29). Public health programs should focus on comprehensive educational campaigns that elevate awareness and understanding of these critical elements, aiming to actively engage individuals in proactive healthcare practices (29). The review provides valuable insights into the risk factors for breast cancer recurrence among postmenopausal women. However, further longitudinal studies are necessary to validate and expand upon these findings. Additionally, future research should explore the interactions between different risk factors and their combined impact on recurrence (30).

To identify and list risk factors for breast cancer recurrence in postmenopausal women, we conducted an extensive literature review and performed detailed data analysis. This was enhanced by expert consultations, collaborative brainstorming sessions, and an iterative review process. We then combined this information to create a thorough overview of established risk factors, which includes:

- Age at diagnosis: Younger age at diagnosis has been linked to a poorer prognosis (31).
- TNM stage, tumor grade, and receptor status: These are major predictive markers for recurrence and influence treatment decisions (31). A high Ki-67 level, indicating rapid cancer cell growth, is also a factor (32).
- Lifestyle factors: Alcohol consumption, smoking, and obesity have been associated with prognosis (31).
- Previous chest radiation: Women who received chest radiation therapy as children or young adults have a significantly increased risk (33).
- Lobular carcinoma *in situ*: Women with LCIS have a substantially increased risk of developing cancer in either breast (34).
- Menstrual history: Early onset of menstruation (before age 12) and/or late menopause (after age 55) slightly increased risk due to prolonged exposure to estrogen and progesterone (32).
- CD44+/CD24- phenotype: This phenotype may be an important factor for malignant relapse after surgery and chemotherapy in invasive ductal carcinoma. Invasive ductal carcinoma is the most common type of breast cancer, and despite advancements in treatment, recurrence within five years after surgery occurs in about 11% of women (35).
- Fear of recurrence: While not a direct cause, studies show that fear of recurrence is associated with psychological distress among cancer survivors (36). One study found that 70% of breast cancer survivors still feared recurrence five years after diagnosis (36).

Breast cancer recurrence in postmenopausal women is influenced by a complex interplay of factors, some like those affecting premenopausal women, while others are unique to this group. Several factors have been identified as potentially contributing to the risk of breast cancer recurrence in postmenopausal women, including:

Hormonal Factors:

- Estrogen and Progesterone Receptor Status: Postmenopausal women with hormone receptor-positive (ER+ and/or PR+) breast cancer are at a higher risk of late recurrence, even up to 10-15 years after initial diagnosis (37). This is because these cancers are fueled by estrogen, which, although lower in postmenopausal women, can still stimulate tumor growth (38). In this study, they discuss the risks associated with hormone therapy containing estrogen and progestins (39). Further elaborates on the link between menopausal hormone therapy and cancer.
- Endogenous Estrogen Levels: Obesity in postmenopausal women is linked to higher levels of endogenous estrogen, which can increase the risk of recurrence (38, 40). This is because fat tissue can convert androgens into estrogen.
- Prior Hormone Replacement Therapy: Past use of HRT, particularly combined estrogen and progestin therapy, has been associated with an increased risk of breast cancer and may also influence recurrence risk (38, 40).

Tumor Characteristics:

- Tumor Size and Grade: Larger tumors and higher-grade tumors (more aggressive) are associated with a greater risk of recurrence, regardless of menopausal status (37).
- Lymph Node Involvement: The presence of cancer cells in the lymph nodes at the time of diagnosis is a strong predictor of recurrence (37).
- HER2 Status: HER2-positive breast cancers tend to be more aggressive and have a higher risk of recurrence, although targeted therapies have improved outcomes (41, 42).

Lifestyle Factors:

- Obesity: As mentioned earlier, obesity increases endogenous estrogen levels, promoting recurrence risk (38, 40). It can also influence insulin levels and other metabolic factors that may play a role.
- Physical Activity: Regular exercise has been shown to reduce the risk of recurrence in some studies (43).
- Diet: A healthy diet may contribute to overall health and well-being, but its direct impact on recurrence risk is still under investigation (43).

Other Factors (32–35):

- Age at Diagnosis: While age itself isn’t a direct cause, older age at diagnosis can sometimes be associated with other factors that influence recurrence risk.
- Comorbidities: Other health conditions, such as diabetes or cardiovascular disease, can sometimes complicate treatment and potentially affect outcomes.

It’s important to note that these factors interact in complex ways, and individual risk profiles vary.

Understanding the epidemiology, clinical characteristics and outcomes of breast cancer recurrence is crucial for enhancing clinical decision-making, optimizing patient management, and ultimately improving the prognosis for this patient population.

## 5 Limitations

Extensive research has underscored the significance and prevalence of recurrences among postmenopausal women with a history of breast cancer. These studies have not only focused on the risk factors and patterns of disease recurrence but have also examined the impact of various treatment modalities and lifestyle factors on the likelihood of cancer recurrence in this patient population. Following an extensive bibliometric review, numerous risk factors were identified as contributing to breast cancer recurrence among postmenopausal women. These factors include advanced age, family history of breast cancer, obesity, and postmenopausal hormone therapy. It is crucial for future studies to prioritize the development of personalized interventions that account for these factors in order to effectively reduce the risk of recurrence. The findings of this review underscore the imperative for sustained and comprehensive research to gain a deeper understanding of the risk factors associated with breast cancer recurrence in postmenopausal women. Given the intricate nature and variability of these factors, it is essential that research endeavors encompass a multidisciplinary approach and foster international collaborations.

## 6 Outlook

The obtained information can contribute to the development of more precise follow-up programs for post-menopausal women, thereby reducing the risk of recurrence. Additionally, the study can enhance awareness of the importance of a healthy lifestyle, inform policy formulation, stimulate new research, and encourage interdisciplinary collaborations within the medical field.

## Data Availability

The original contributions presented in the study are included in the article/supplementary material. Further inquiries can be directed to the corresponding author.
